# Association between breastfeeding duration and BMI, 2009–2018: a population-based study

**DOI:** 10.3389/fnut.2024.1463089

**Published:** 2024-09-04

**Authors:** Jiaqing Sun, Jian Han, Xiaofeng Jiang, Yali Ying, Shenghao Li

**Affiliations:** ^1^Wenyan Branch of the First People’s Hospital of Xiaoshan District, Hangzhou, China; ^2^The First People’s Hospital of Xiaoshan District, Hangzhou, China

**Keywords:** breastfeeding, BMI, obesity, children, NHANES

## Abstract

**Background:**

In the 21st century, childhood overweight and obesity have become major public health issues worldwide. Previous studies have shown that breastfeeding helps prevent overweight or obesity in children. Despite the significant advantages of breastfeeding, the global exclusive breastfeeding rate for infants under 6 months old is only 40%, while in the United States, the rate is only 25%. The aim of this study is to explore the relationship between breastfeeding duration and BMI in children aged 2 to 6 in the United States, and to raise awareness of breastfeeding.

**Methods:**

A cross-sectional study included 2,769 participants between the ages of 2 and 6 from a sample that represented the entire NHANES 2009–2018. Data was analyzed using EmpowerStats, (www.empowerstats.com) linear regression as well as Chi-square test, t-tests, multivariate regression analysis and smooth cure fitting were done.

**Results:**

Breastfeeding duration long-term group exhibited a statistically significant negative association with BMI, with a regression coefficient of −0.21 (*P* < 0.05). The continuous analysis of breastfeeding duration by tertile also demonstrate a statistically significant negative association with BMI. Subgroup analysis revealed that the potential benefits of breastfeeding on BMI were more obvious in low-income environments and maternal age 18 to 35 years, with a regression coefficient of −0.57 and −0.24, respectively (all *P* < 0.05).

**Conclusion:**

The findings emphasize the importance of breastfeeding in reducing childhood overweight/obesity and preventing associated diseases, both in clinical and public health settings.

## 1 Introduction

In the 21st century, childhood overweight and obesity have emerged as significant public health challenges in many developed countries (1–3), and posing serious health threats globally (4). In 2022, the World Health Organization (WHO) reported that around 37 million children under the age of 5 are obese, with over 390 million children and teenagers aged 5 to 19 also being overweight. In addition, estimated 160 million children suffer from obesity (5). The global incidence of childhood obesity is increasing, and it is expected that by 2030, the numbers of overweight and obese individuals will reach 40 million and 254 million, respectively (6). In the past forty years, the mean Body Mass Index(BMI) for American adults between 18 and 25 years old increased from 23.0 to 27.5 (95% CI: 22.8–23.2), while the worldwide rate of adult obesity doubled, highlighting the enduring effects of childhood obesity (7). Children who are overweight or obese are likely to continue being overweight or obese as adults (3, 8), making early overweight and obese a strong predictor of adult overweight and obesity (6). Overweight and obesity might lead to a series of serious health problems, which may appear in the short or long-term, including type 2 diabetes, cardiovascular problems (3, 8), increased mortality, premature death, disability (6, 8), and mental health problems (9). Childhood obesity has both health and economic impacts, leading to expenses like medical costs and reduced productivity due to increased absenteeism (10).

Breastfeeding provides many advantages for infants and young children, and is widely regarded as the optimal nutrition source (11). Breast milk has the effects of promoting somatic growth, regulating postnatal intestinal functions, boosting immunity, and supporting brain development (12). Breast milk is acknowledged for its role as the primary source of nourishment for infants, offering ideal nutrition, boosting the immune system, and fostering special connections between mother and child. The WHO, the American Academy of Pediatrics, and the American College of Obstetricians and Gynecologists have all endorsed the suggestion of exclusive breastfeeding for the initial six months of a baby’s life (11, 13, 14). The WHO also recommends that breastfeeding should continue even after adding complementary foods, until the child is two years old or older, in order to achieve optimal growth, development, and health (14).

In Singapore, a cohort study discovered that children breastfed for less than four months had a 0.18 (−0.01, 0.38) (β (95% CI) increase in BMI at age 6 compared to those breastfed for over four months, after adjusting for confounding factors, accompanied by a total of 1.83 mm (0.05, 3.61) (β(95% CI) of larger skin folds (15). A cohort study of low-income Mexican/Mexican American mothers revealed significant differences between breastfed and formula-fed children under 3 years of age (*F* = 4.644, *P* < 0.05), and breastfed children were 18% less likely to be obese compared to their formula-fed counterparts (16). In the United States, a research project examined children aged 4 to 8 and discovered a dose-response relationship between how long they were breastfed and the likelihood of developing obesity in early childhood. Exclusive breastfeeding at 6 months reduced the risk of obesity by 60% compared to non-breastfeeding (95% CI: 0.18–0.91) (17).

Early breastfeeding in children has a potential correlation with overweight and obesity. Despite the clear advantages of breastfeeding, the worldwide rate of exclusive breastfeeding for infants under 6 months is just 40% (18). In a 2020 study conducted by the Centers for Disease Control and Prevention in the U.S., it was found that just 25% of infants are advised to exclusively breastfeed until they reach six months of age (19). The low breastfeeding rate makes research on breastfeeding more urgent. In addition, recent research has mainly focused on the impact of overweight and obesity on older children and adolescents, with less research on children aged 2 to 6 years old. The age range of 2 to 6 is a period of rapid physical, cognitive, and social development for a child. The developments that have occurred in these four years will affect children’s lives in various ways in the future. The objective of this research is to investigate the impact of the length of breastfeeding on the BMI of children between 2 and 6 years old in the U.S. The significance of this study is multifaceted. Firstly, it helps more and more literature explore the long-term effects of early nutrition on health, with a particular focus on how breastfeeding affects BMI trajectory. Secondly, by utilizing extensive and detailed NHANES datasets, this study provides strong epidemiological analysis that helps clarify inconsistencies and gaps in existing studies. Finally, the results of this research could offer evidence-supported suggestions for breastfeeding techniques and influence efforts to prevent obesity beginning in early childhood.

## 2 Materials and methods

### 2.1 Study population and design

NHANES is a nationally representative comprehensive cross-sectional study designed to assess the health and nutritional status of the adult and child populations in the United States (20). The survey consists of questions about demographics, dietary habits, socioeconomic status, and health factors (21). The research procedures were all authorized by the National Center for Health Statistics Ethics Review Board, and informed consent was obtained from all participants prior to data collection (22). For more detailed information, please visit the official website.^1^

**FIGURE 1:**
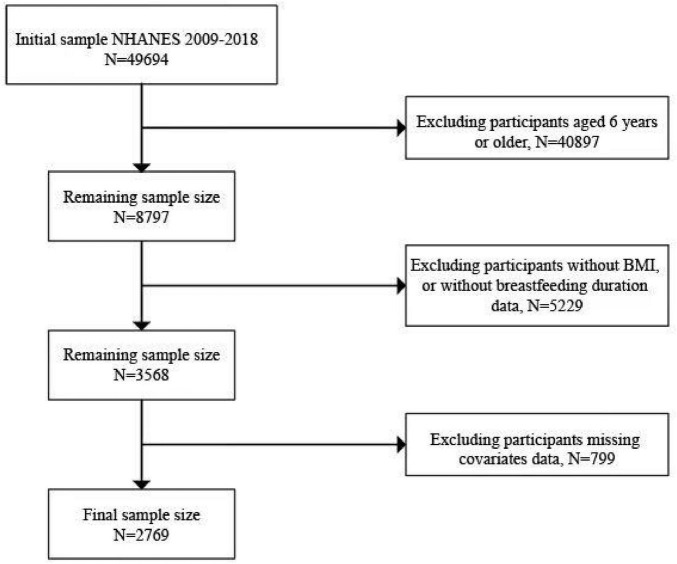
Flowchart of sample selection from the NHANES 2009–2018. We included a total of 49,694 samples, and after excluding variables with incomplete data, such as missing BMI, breastfeeding duration, and other covariates, we screened a total of 2,769 samples.

A total of 2,769 individuals aged 2 to 6 years were involved in this survey, which was part of a comprehensive NHANES 2009–2018 study. We excluded 40,897 participants who did not meet the age criteria from the study sample. We further excluded 3,793 participants with missing BMI data or 1,436 participants with incomplete breastfeeding duration samples. In addition, 799 participants missing co-variates data were excluded, leaving a total of 2,769 participants in the present analysis. The detailed screening process is illustrated in Figure 1.

### 2.2 Assessment of BMI (outcome)

The article includes details about the weight status categories and percentiles for BMI-for-age, which are based on Centers for Disease Control and Prevention (23). This information highlights the categorical representation of the BMI variable, providing a standardized method for assessing weight status in children and teenagers. The weight status categories are divided into four distinct groups: underweight, healthy weight, overweight, and obesity. These categories are determined based on the percentile range of the BMI-for-age measurements. The specific percentiles for each category are as follows in Table 1.

**TABLE 1:** The weight status categories. Reproduced from (60).

Weight status category	Percentile range
Underweight	Less than the 5th percentile
Healthy Weight	5th percentile to less than the 85th percentile
Overweight	85th to less than the 95th percentile
Obesity	Equal to or greater than the 95th percentile

### 2.3 Assessment of breastfeeding duration (exposure)

We define breastfeeding as any form of breastfeeding that is reserved for direct breast feeding or consumption of extruded breast milk, and was not limited to exclusive breastfeeding. The timing of when solid food was introduced was assessed by asking questions raised at 3 and 9 months (2). Introducing solid foods might influence on the changes in breastfeeding patterns for infants and young children, so the breastfeeding duration in this study is set at 3 and 9 months as critical points. Participants were asked to provide information on the duration of breastfeeding (in days) and were divided into three groups depending on the duration: short-term (0 ≤ days < 90), medium-term (90 ≤ days < 270), and long-term ( ≥ 270 days).

### 2.4 Covariates assessment

Information on age, maternal age at conception, sex, birthweight, maternal smoking status, and ethnicities was self-reported. The classification of ethnicities included Mexican American, other Hispanic, non-Hispanic White, non-Hispanic Black, and other ethnicities, such as multi-ethnical individuals (24, 25). Physical activity was divided into 2 groups: inactive (the number of days with at least 60 minutes of physical activity per day was ≤ 3 days in the past 7 days), active (≥ 4 days). According to the annual family income questionnaire, income level was divided into 3 groups: Under $20,000, $20,000 to 75,000 and Over $75,000. Divide into two groups based on whether smoking during pregnancy: never smoker and smoker (26).

### 2.5 Statistical analysis

The study population’s baseline characteristics were evaluated using a weighted linear regression model and weighted Chi-square test. The study utilized Multiple Regression analysis to evaluate the relationship between the length of breastfeeding and BMI as a continuous variable. Smooth curve fitting was utilized to examine the correlation between the length of breastfeeding and BMI. All analytical processes were performed by using EmpowerStats^2^ version 5.0, with a significance threshold set at a *P* < 0.05. Additionally, to mitigate large fluctuations in the dataset, we employed a weighting strategy.

## 3 Results

### 3.1 Baseline characteristics

This article included 2,769 participants (1410 male and 1359 female) from NHANES 2009–2018, the mean(SD) age was 3.76 (1.4) years; the gender ratio is balanced, with 50.92% of people were males. Compared with the lowest tertile of breastfeeding duration, higher tertile groups were more likely to be elder, mothers give birth to children at a much older age, non-Hispanic White, income over $75,000, and mother never smoked when pregnant (all *P* < 0.05). Highest tertile group has the highest number of people with a healthy weight (*P* < 0.05). The detailed characteristics of participants were shown in Table 2.

**TABLE 2:** Baseline characteristics of the study population.

Characteristic	Tertile of breastfeeding duration(N = 2,769)			*P*-value
	Short-term	Medium-term	Long-term	
Age, years	3.77 ± 1.34	3.88 ± 1.38	3.90 ± 1.33	0.1110
Maternal age at conception, years	26.77 ± 6.28	28.01 ± 5.83	30.01 ± 5.51	< 0.0001
Birthweight, pounds	6.84 ± 1.31	6.76 ± 1.23	7.08 ± 1.21	< 0.0001
BMI, (kg/m^2^)	16.50 ± 2.06	16.49 ± 1.99	16.22 ± 1.75	0.0012
BMI				0.0019
Underweight	23 (3.10)	47 (4.35)	26 (2.70)	
Healthy weight	394 (54.62)	568 (52.82)	552 (56.78)	
Overweight	179 (24.78)	236 (21.98)	252 (25.93)	
Obesity	126 (17.50)	224 (20.85)	142 (14.59)	
Sex				0.4356
Male	376 (52.09)	555 (51.59)	479 (49.30)	
Female	346 (47.91)	520 (48.41)	493 (50.70)	
Ethnicities				< 0.0001
Mexican American	123 (17.20)	190 (17.66)	140 (14.42)	
Other Hispanic	62 (8.50)	103 (9.59)	67 (6.93)	
Non-Hispanic White	381 (52.76)	550 (51.21)	590 (60.71)	
Non-Hispanic Black	93 (12.89)	124 (11.52)	70 (7.17)	
Other Ethnicities - Including Multi-Ethnical	63 (8.64)	108 (10.02)	105 (10.78)	
Annual family income				< 0.0001
Under $20,000	159 (21.99)	206 (19.13)	121 (12.42)	
20,000 to 75,000	383 (53.01)	499 (46.42)	426 (43.81)	
Over $75,000	180 (25.00)	370 (34.45)	425 (43.77)	
Physical activity				0.0610
Inactive	42 (5.82)	93 (8.68)	83 (8.57)	
Active	680 (94.18)	982 (91.32)	889 (91.43)	
Maternal smoking status				< 0.0001
Smoker	82 (11.42)	86 (7.99)	33 (3.41)	
Never smoker	640 (88.58)	989 (92.01)	939 (96.59)	

For categorical variables, *P*-value was calculated by the weighted chi-square test. For continuous variables, weighted linear regression model was used to calculate *P*-value.

### 3.2 Association between breastfeeding duration and BMI

Compared to the lowest tertile of breastfeeding duration, the middle tertile had a non-significant association with BMI, while the highest tertile exhibited a statistically significant negative association with BMI, with a regression coefficient of −0.21 (*P* < 0.05). The continuous analysis of breastfeeding duration by tertile also demonstrate a statistically significant negative association with BMI. These findings suggest that a longer duration of breastfeeding may be associated with a lower BMI in the population studied, as shown in Table 3.

**TABLE 3:** Regression Coefficients (β) in the association between breastfeeding duration and BMI on a continuous scale.

Exposure	Non-adjusted model			Adjust I model			Adjust II model		
	β	OR (95%CI)	*P*	β	OR (95%CI)	*P*	β	OR (95%CI)	*P*
Breastfeeding duration	−0.00	(−0.00, −0.00)	0.0012	−0.00	(−0.00, −0.00)	0.0044	−0.00	(−0.00, −0.00)	0.0104
**Tertile of breastfeeding duration**									
Short-term		1 (ref)			1 (ref)			1 (ref)	
Medium-term	−0.02	(−0.20, 0.17)	0.8723	−0.02	(−0.20, 0.17)	0.8554	0.03	(−0.15, 0.22)	0.7272
Long-term	−0.29	(−0.47, −0.10)	0.0024	−0.25	(−0.43, −0.06)	0.0091	−0.21	(−0.39, −0.02)	0.0305
*P* for trend	−0.15	(−0.24, −0.06)	0.0011	−0.13	(−0.22, −0.04)	0.0051	−0.11	(−0.21, −0.02)	0.0172

Non-adjusted model: none. Adjust I model: ethnicities and gender-adjusted. Adjust II model: ethnicities, gender, age, income, physical activity, mother age, mother smoking status, and birthweight-adjusted.

**FIGURE 2:**
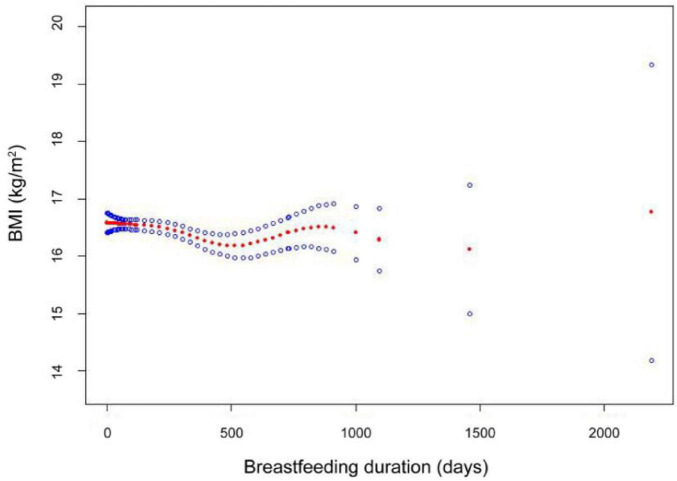
The smooth-fitting curves between breastfeeding duration and BMI. Smooth curve fitting results indicate that the breastfeeding duration is non-linear associated with BMI among participants. Adjust model: ethnicities, gender, age, income, physical activity, mother age, mother smoking status, and birthweight-adjusted.

In addition, the results of the smooth-fitting curves further confirmed the non-linear correlation between BMI and the duration of the breastfeeding period, as shown in Figure 2. The breastfeeding duration days (in red) with 95% CIs (in blue) determined using the generalized additive model. Within the range of 0 days to approximately 1000 days, we can observe a slight U-shaped trend. The BMI value slightly decreases during the early stages of breastfeeding, then reaches its lowest point around 500 days, and then slowly increases. After a longer feeding period, the relationship becomes less clear.

### 3.3 Subgroup analyses

We conducted a subgroup analysis of annual family income level. The detailed content is shown in Table 4. For households with annual incomes below $20,000, we analyzed the relationship between breastfeeding duration and BMI. Compared to the reference group (breastfeeding duration < 90 days), the middle-duration group showed no significant correlation with BMI. However, the longest-duration group exhibited a statistically significant negative correlation with BMI, with a regression coefficient of −0.57 (*P* < 0.05). The continuous analysis of breastfeeding duration by tertile also demonstrate a statistically significant negative association with BMI. The above conclusion is consistent with the conclusion when subgroup analysis was not conducted. However, analysis of subgroup with an annual family income between $20,000 and $75,000, as well as subgroup above $75,000, showed no statistical correlation between breastfeeding duration and BMI.

**TABLE 4:** Regression Coefficients (β) in the association between breastfeeding duration and BMI in annual family income under $20,000 on a continuous scale.

Exposure	Non-adjusted model			Adjust I model			Adjust II model		
	β	OR (95%CI)	*P*	β	OR (95%CI)	*P*	β	OR (95%CI)	*P*
Breastfeeding duration	−0.00	(−0.00, −0.00)	0.1068	−0.00	(−0.00, −0.00)	0.0544	−0.00	(−0.00, −0.00)	0.0529
**Tertile of breastfeeding duration**									
Short-term		1 (ref)			1 (ref)			1 (ref)	
Medium-term	−0.15	(−0.54, 0.25)	0.4667	−0.16	(−0.55, 0.24)	0.4369	−0.18	(−0.57, 0.22)	0.3827
Long-term	−0.46	(−0.89, −0.02)	0.0392	−0.50	(−0.94, −0.07)	0.0235	−0.57	(−1.01, −0.12)	0.0130
*P* for trend	−0.23	(−0.44, −0.01)	0.0410	−0.25	(−0.46, −0.03)	0.0250	−0.28	(−0.50, −0.06)	0.0143

Non-adjusted model: none. Adjust I model: ethnicities and gender-adjusted. Adjust II model: ethnicities, gender, age, physical activity, mother age, mother smoking status, and birthweight-adjusted.

35 years old is a threshold for female fertility. After 35 years old, women’s fertility potential gradually decreases, and the number and quality of primordial follicles in oocytes decrease (27). In our subgroup analysis, we divided them into three groups: under 18 years old, between 18 and 35 years old, and 35 years old and above. We conducted subgroup analysis according to the age of the mother at the time of delivery, as shown in Table 5. The analysis of mothers with delivery age between 18 and 35 years showed that breastfeeding was a protective factor for BMI, with a regression coefficient of −0.24 (*P* < 0.05). A continuous analysis of the duration of breastfeeding in three equal groups also showed that there was a statistically significant negative correlation between the duration of breastfeeding and BMI. The above conclusions were consistent with those without subgroup analysis. However, this conclusion was not found in the group with delivery age less than 18 years or age equal to or older than 35 years.

**TABLE 5:** Regression Coefficients (β) in the association between breastfeeding duration and BMI in maternal age during delivery 18–35 years old on a continuous scale.

Exposure	Non-adjusted model			Adjust I model			Adjust II model		
	β	OR (95%CI)	*P*	β	OR (95%CI)	*P*	β	OR (95%CI)	*P*
Breastfeeding duration	−0.00	(−0.00, −0.00)	0.0116	−0.00	(−0.00, −0.00)	0.0285	−0.00	(−0.00, −0.00)	0.0187
**Tertile of breastfeeding duration**									
Short-term		1 (ref)			1 (ref)			1 (ref)	
Medium-term	−0.04	(−0.25, 0.16)	0.6807	−0.05	(−0.26, 0.16)	0.6459	−0.02	(−0.22, 0.18)	0.8461
Long-term	−0.27	(−0.47, −0.06)	0.0130	−0.23	(−0.44, −0.03)	0.0277	−0.24	(−0.45, −0.03)	0.0242
*P* for trend	−0.14	(−0.24, −0.03)	0.0092	−0.12	(−0.23, −0.02)	0.0218	−0.13	(−0.23, −0.02)	0.0175

Non-adjusted model: none. Adjust I model: ethnicities and gender-adjusted. Adjust II model: ethnicities, gender, age, physical activity, mother age, mother smoking status, and birthweight-adjusted.

## 4 Discussion

In this cross-sectional study of 2,769 children aged 2 to 6 in the U.S., we found that children who breastfed for a longer period of time were less likely to be overweight or obese at the age of 2 to 6. The observed associations were independent of major lifestyle risk factors, self-reported lifestyle habits, and socio-demographic factors. One possible explanation is that formula fed infants always overeat (28), compared to breastfeeding, formula feeding can lead to faster growth in infancy (29, 30). Formula-fed infants are typically bottle-fed (31), which may lead to poor self-regulation of intake and reduced prolonged satiety (32). Mothers who bottle-feed their infants often have greater control over their baby’s intake, potentially leading to practices that pressure infants to consume more, such as encouraging them to finish the bottle (33). This may hinder the development of infants’ ability to self-regulate their intake, leading to overfeeding, poor satiety response, and excessive weight gain (34). Compared to children who are directly breastfed, children who are breastfed through bottles are 67% less likely to experience high satiety, and early direct breastfeeding is associated with better appetite regulation in later childhood (32). Breastfeeding facilitates infant self-regulation of milk intake, as mothers have limited control over the process, while formula feeding, often managed by nursing staff, may be more passive, potentially hindering infants’ ability to self-regulate intake (34). Another possible explanation is that there are differences in the composition between breast milk and formula milk. While infant formula is primarily derived from bovine milk, its composition differs significantly from human milk in terms of fats, proteins, vitamins, and minerals (12). Formula milk contains higher protein content than breast milk, with bovine milk protein concentrations ranging from 1.80 to 2.0 g/L (35). Research suggests that increased protein intake during infancy correlates with accelerated weight gain and elevated obesity risk in later life (35, 36). Bovine milk fat globule membrane (MFGM) has demonstrated beneficial effects on infant development (37, 38), leading to its inclusion in most infant formulas (39). As cholesterol is a component of MFGM, infant formula contains higher concentrations of milk cholesterol compared to human milk (40). Hypercholesterolemia, a common lipid abnormality in obesity, often results from lipid metabolism disorders associated with overweight and obesity. These disorders can lead to elevated levels of low-density lipoprotein (LDL) and total cholesterol in the body. These findings emphasize the potential clinical and public health significance of adhering to breastfeeding as a way to reduce childhood overweight or obesity and prevent related diseases. The association between longer breastfeeding and reduced early childhood obesity observed in our study is consistent with previous clinical trial results in children and adolescents. In a large-scale cross-sectional study conducted in Qingdao, China. Research has found that children who breastfeed for more than a year have a lower risk of overweight and obesity, especially boys aged 9 to 11 (41). A study by Croatian on second and third grade student groups showed that breastfeeding for more than 6 months is a protective factor for overweight and obesity (42). In addition, a cross-sectional survey study in Shanghai showed that exclusive breastfeeding is associated with a reduced risk of central obesity (RR:0.76; 95% CI: 0.60, 0.96, *P* < 0.05) (43).

Curve fitting analysis revealed a complex, non-linear relationship between breastfeeding duration and children’s BMI, suggesting an optimal range of breastfeeding duration associated with lower BMI. This complex, non-linear relationship aligned with previous research findings. Harder found a non-linear dose-response relationship between breastfeeding and later obesity risk (44), while Rito emphasized the complex, multifactorial nature of this relationship (45). We observed a slight decrease in BMI during the first 500 days of breastfeeding, potentially reflecting its protective effect against rapid weight gain in infants and young children. The review by Woo Baidal suggested that early life nutritional interventions, particularly breastfeeding, may play a key role in preventing childhood obesity (46). In addition, Ong and Loo’s research suggested that breastfeeding may reduce the risk of obesity in the future by regulating early growth rate (47). The BMI value reaches its nadir at approximately 500 days (16.5 months) of breastfeeding before gradually increasing. This trend may be attributed to changes in dietary behavior after 18 months, as children transition from complementary foods to adult-like dietary patterns, potentially impacting their BMI. Emmett and Jones found that between 18 and 24 months, children begin consuming foods similar to family members, potentially increasing exposure to energy-dense foods and affecting BMI (48). Nicklaus emphasized that 18–24 months is a critical period for dietary behavior formation, with children adopting family diets, potentially leading to increased energy intake and subsequent BMI changes (49).

In our study, we examined the relationship between the duration of breastfeeding and BMI at different annual family income levels. The results of subgroup analysis revealed that the potential benefits of breastfeeding on BMI are more pronounced in low-income environments. However, this association was not observed in the two subgroups with higher annual income (from $20000 to $75000 and above). In social epidemiology and public health research, income has been recognized as a factor that increases the risk of developing subsequent obesity (50). Research showed that in developed countries, lower social classes are associated with a higher risk of obesity, which even affects infants and young children (51). This may be because babies born in impoverished backgrounds are more likely to be exposed to poor breastfeeding practices (51). Low socio-economic status is associated with high parental pressure and restrictive feeding practices (39), which may lead to overfeeding. In addition, high-income families are more likely to acquire knowledge about nutrition and have more opportunities to engage in other behaviors that help maintain a healthy BMI, thereby reducing the relative impact of breastfeeding duration.

Long-term breastfeeding is not only a protective factor for obesity in children and adolescents, but also a protective factor for obesity in adults (52–54). Previous studies by others have shown that breastfeeding protects from diarrhea and respiratory infections, seasonal allergic rhinitis, and diabetes (55–57). Overall, these findings contribute to the growing body of evidence supporting the multifaceted influence of breastfeeding duration, socio-demographic factors, and lifestyle variables on BMI outcomes in early childhood. The implications of these results extend to public health interventions aimed at promoting breastfeeding and addressing disparities in BMI outcomes across diverse demographic groups. Future research should continue to explore the longitudinal effects of breastfeeding duration on BMI and elucidate the underlying mechanisms driving these associations to inform targeted interventions and policies aimed at improving childhood obesity prevention and management.

This article has some limitations, including the overall small sample size might result in bias, particularly in certain ethnic categories, in which the smallest number of participants are included. Then, the study did not delve into the impact of mixed feeding on BMI. It is important to take into account these restrictions when analyzing the findings of a study. Furthermore, due to incomplete information in the NHANES database, several important covariates could not be included in our analysis. These include maternal body mass index, maternal weight fluctuations before and during pregnancy, gestational diabetes, and mode of delivery. The absence of these factors may introduce potential bias to our findings. A limitation of our study is the modest practical significance of our findings, despite achieving statistical significance. The regression coefficients exhibit relatively small effect sizes, despite being statistically significant. This may be attributed to our sample size which, although substantial, might not have been sufficient to detect more pronounced practical effects. We also observed wide confidence intervals in several instances, some of which included zero. This pattern indicates uncertainty in our estimates and potentially constrains the practical implications of our results. These limitations necessitate cautious interpretation of our findings and emphasize the need for future studies with larger sample sizes to more accurately estimate the relationship between breastfeeding duration and BMI. Lastly, this article was only based on the database of the U.S. and has not surveyed the entire population worldwide, so it might not be applicable to developing countries.

An interesting result of our study is that we found no correlation between breastfeeding duration and BMI in the maternal age group more than 35 years old and above (*P* > 0.05). This may be related to the presence of other confounding factors. Research conducted in Taiwan revealed that mothers’ BMI (β = 0.240; *P* < 0.05), and education level (β = −0.141; *P* < 0.05) are important factors affecting children’s BMI, and together with monthly family income, it explains an additional 8% variance in children’s BMI, which is statistically significant (58). Obesity is also influenced by many other factors, such as child gender (59), parental inheritance, etc., (50). In addition, there was no correlation between breastfeeding and BMI in the group under 18 years old (*P* > 0.05). We believe this may be due to the strong support of American families and the low number of young mothers. Only 3.47% of the respondents were mothers under the age of 18.

## 5 Conclusion

The research suggests that the correlation between the duration of breastfeeding and early childhood BMI outcomes. These findings emphasize the potential clinical and public health implications of adhering to breastfeeding as a way to reduce childhood overweight or obesity and prevent related diseases. This conclusion is particularly evident in households with an annual income of less than $20,000 and maternal aged between 18 and 35. It is necessary to conduct larger scale prospective cohort studies to further replicate this finding and elucidate the potential role of extending breastfeeding time in reducing overweight and obesity in young children.

## Data Availability

Publicly available datasets were analyzed in this study. This data can be found here: https://www.cdc.gov/nchs/nhanes/.

## Footnotes

1
https://www.cdc.gov/nchs/nhanes/

2
www.empowerstats.com
